# Lamb Wave-Based Structural Health Monitoring on Composite Bolted Joints under Tensile Load

**DOI:** 10.3390/ma10060652

**Published:** 2017-06-14

**Authors:** Bin Yang, Fu-Zhen Xuan, Yanxun Xiang, Dan Li, Wujun Zhu, Xiaojun Tang, Jichao Xu, Kang Yang, Chengqiang Luo

**Affiliations:** 1 School of Mechanical and Power Engineering, East China University of Science and Technology, Shanghai 200237, China; yangbin@ecust.edu.cn (B.Y.); yxxiang@ecust.edu.cn (Y.X.); wjzhu@mail.ecust.edu.cn (W.Z.); 18767166026@163.com (J.X.); yangkangcxm@163.com (K.Y.); chengqiangLuo@126.com (C.L.); 2 The Department of Electronic Engineering, Fudan University, Shanghai 200433, China; lidan@fudan.edu.cn; 3 Beijing Spacecrafts, China Academy of Space Technology, Beijing 100094, China; tangxiaojun2214@163.com

**Keywords:** Lamb wave propagation, composite bolted joints, structural health monitoring, FE simulation

## Abstract

Online and offline monitoring of composite bolted joints under tensile load were investigated using piezoelectric transducers. The relationships between Lamb wave signals, pre-tightening force, the applied tensile load, as well as the failure modes were investigated. Results indicated that *S0*/*A0* wave amplitudes decrease with the increasing of load. Relationships between damage features and *S0*/*A0* mode were built based on the finite element (FE) simulation and experimental results. The possibility of application of Lamb wave-based structure health monitoring in bolted joint-like composite structures was thus achieved.

## 1. Introduction

The need for stronger and lighter structures in the fields of aerospace, ship engineering, and the automotive industry has driven the use of composite structures. Even though the structure length can be as long as a hundred meters, the maximum dimension of an integrative composite structure is limited into approximately 20 m in length by the manufacturing process, such as the commonly used resin transfer molding (RTM) method [1,2]. Therefore, bolted joints between composite components are necessary when manufacturing large structures [3,4,5]. However, under real service conditions, damages, e.g., debonding and delamination, in the composite bolted joints are frequently initiated from the fastener holes, which further degenerate structural reliability [6]. Therefore, investigations on the development and optimization of quantitative damage detection and characterization in complex bolted joints are essential.

Structural health monitoring (SHM) is defined as nondestructive and continuous monitoring of structures. SHM can identify and monitor the developing defects before their degeneration into a failure [7,8]. In recent years, guided wave-based technology has been shown to be an effective SHM method, attributed to its high sensitivity and versatility [9]. Guided wave can be generated by piezoelectric transducers on the studied structure. This transducer is very light, thin, and can be used as actuator and sensor, respectively [10]. Moreover, guided waves are sensitive to small-scale damages and could propagate over long distance [11]. The Lamb wave is one of the guided wave modalities. The Lamb wave propagates by particle motions between the two surfaces in a thin plate-like medium [12]. Using Lamb wave-based SHM systems, there is no need to scan the entire object under consideration, and all of the data can be acquired from a single probe position. Even though detection and localization of defects in simple thin-walled composite laminates have been studied extensively [13,14,15], research on the inspection of complex composite assemblies using Lamb wave-based systems is still insufficient.

From the mechanical point of view, most researchers are concerned about the geometrical influence on the bolts of the composite in different composite types with various fiber stacking sequences [16,17,18,19,20]. Researchers claim that the failure modes of composite bolted joints are strongly dependent on the base materials and the designed joint styles. For instance, Zhou et al. [16] investigated the geometric parameter influence on the failure mode of composite single lap bolted joints. The failure modes of glass fiber-reinforced plastic bolted laminates were also extensively studied by Feo et al. [17,18,19]. Ahmad et al. [20] was concerned with the fracture characteristics in composite double lap bolted joints, and found that the joint finally failed by net-tension damage. Meanwhile, researchers in the mentioned references also found that the structural integrity of the composite joints was threatened severely by the harsh environment during operating life. The joints may result in sharp failure with little advanced warning [21]. Therefore, it is necessary to develop SHM techniques that focus on continuous/real-time monitoring of composite bolted joints. Recently, guided wave technology has been successfully employed for damage detection in bonded joints [22,23,24,25,26,27]. Mohammad et al. [22] evaluated the integrity of a composite skin-stringer joint using the scattering behavior of Lamb waves. Baiyang et al. [23] used ultrasonic guided waves to investigate the adhesive bonds between composite laminates. Dulip et al. [24] developed a wavelet spectral finite element model for studying transient dynamics and wave propagation features in adhesively bonded composite joints. Guided wave propagation characteristics in bonded composite structures were also investigated by Deng et al. [25]. References devoted to using guided waves to detect defects in the other composite joint types such as T-joint [26] and L-joint [27] were also found. However, very few references concerned the application of Lamb wave-based SHM technology to monitor the damages in composite bolted joints.

The major emphasis of this paper was placed on the inspection of composite bolted joints based on the Lamb wave propagation phenomenon. The bolted joints of interest were composed of two cases with different joint length-to-width ratios. The captured Lamb wave signals in the joints before, during and after tensile test were investigated. Lamb wave structures in the researched joints were analyzed, and the relationships between Lamb wave signals, pre-tightening force, the applied tensile load, as well as the failure modes were discussed. Lamb wave propagation behavior in the joints especially in the overlapped jointed region was analyzed using a 3D finite element simulation. All the input parameters in the simulation were determined experimentally by fundamental mechanical tests.

## 2. Experimental Details

### 2.1. Materials and Processing

**Figure 1:**
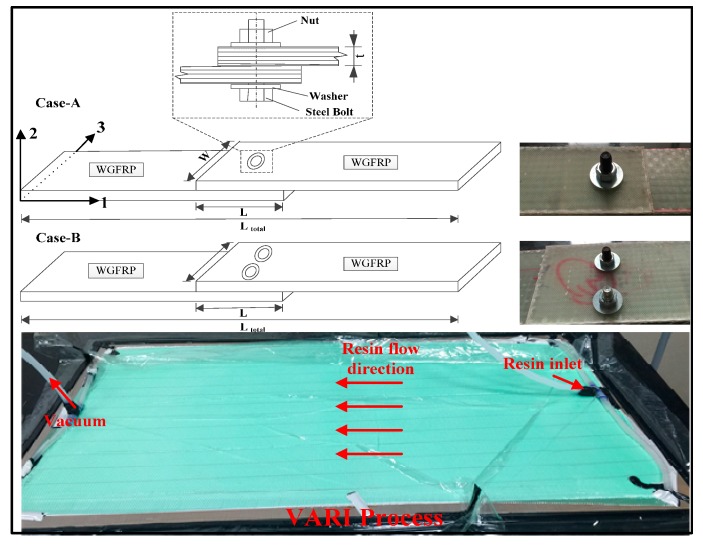
Schematic diagram of the vacuum-assisted resin injection (VARI) manufacturing process and the two studied composite bolted joints with the coordinate system.

The polymer used in the composites as the matrix was vinyl ester resin. The resin can be cured at room temperature in the presence of a hardening agent and an accelerating agent. The hardening agent was methyl ethyl ketone peroxide (MEKP), and the accelerating agent was dimethylaniline. The resin was mixed with hardening agent and accelerating agent at mass ratio of 1:2:0.5. The reinforced material was plain woven glass fabric cloth (WGF). We adopted a vacuum-assisted resin injection (VARI) process to manufacture the WGF/epoxy composites in the bolted joints. Figure 1 shows the schematic diagram of the VARI process. During the manufacturing process, we used a glass plate at the bottom of the VARI system as a holder. After coating the release cloth on the holder, we placed four layers of woven glass fabric. The stacking sequence of the plate was [90°/0°]_4_. After the system was covered with peel and silk ply, the system was closed by a vacuum bag and sealant tape. We vacuumed the system at 600 mbr at room temperature for 10 h to ensure complete vacuum conditions. To ensure the resin flowing uniformly, a delivery pipe was fixed at the entrance. After resin injection and curing for 24 h, the composites were demolded. The final dimension of the manufactured WGF/epoxy composite laminates was 800 × 800 × 2 mm^3^, and we cut it into the required dimension by a low speed diamond saw blade cutting machine under water protection.

The fastener holes on WGF/epoxy composite laminates were made by an electric drill, and the diameter of the holes were 10 mm. It should be noted that the drill speed was set to very slow, and initial damage was not formed after the drilling operations. Two composite bolted joint cases were investigated: Case A were composite joints with a single bolt, while the joints in Case B had two steel bolts in them. As shown in Figure 1 and listed in Table 1, each case included three categories of joints with the jointed length (L) to width (W) ratio (L/W) various from 1:1 to 3:1. Meanwhile, the coordinate system adopted in the following work was given in Figure 1. The total length and the bolt locations in the joints were listed in Table 1.

### 2.2. Fundamental Mechanical Tests

In order to determine the stiffness and strength parameters of WGF/epoxy composites in the bolts, fundamental mechanical tests that included 90°, 0° and ±45° tensile and compression tests were accomplished using an INSTRON-4505 servo-electric testing machine (Instron, Grove City, PA, USA). The nominal maximum force of the adopted test machine was 500 kN. The dimensions of the test specimens were in accordance with the American Society for Testing Materials (ASTM) standard. Specially, to evaluate the interfacial performance between adjacent glass fabric cloth layers, we tested the interlaminate performance of the composites by double cantilever beam (DCB), three-point end notched flexural (3ENF), and Mode III fracture toughness tests, respectively. It should be noted that a pre-crack with a length of 25 mm was laid in the middle thickness of each specimen. The used mechanical testing standards, specimen’s dimension, and the calibrating length of different tests were listed in Table 2. All of the mentioned mechanical tests were performed at room temperature. The cross-head speed was set as 2 mm/min, and five specimens were tested per type to calculate their average value.

**Figure 2:**
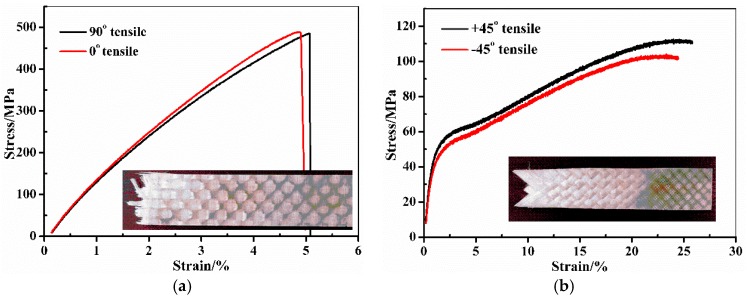

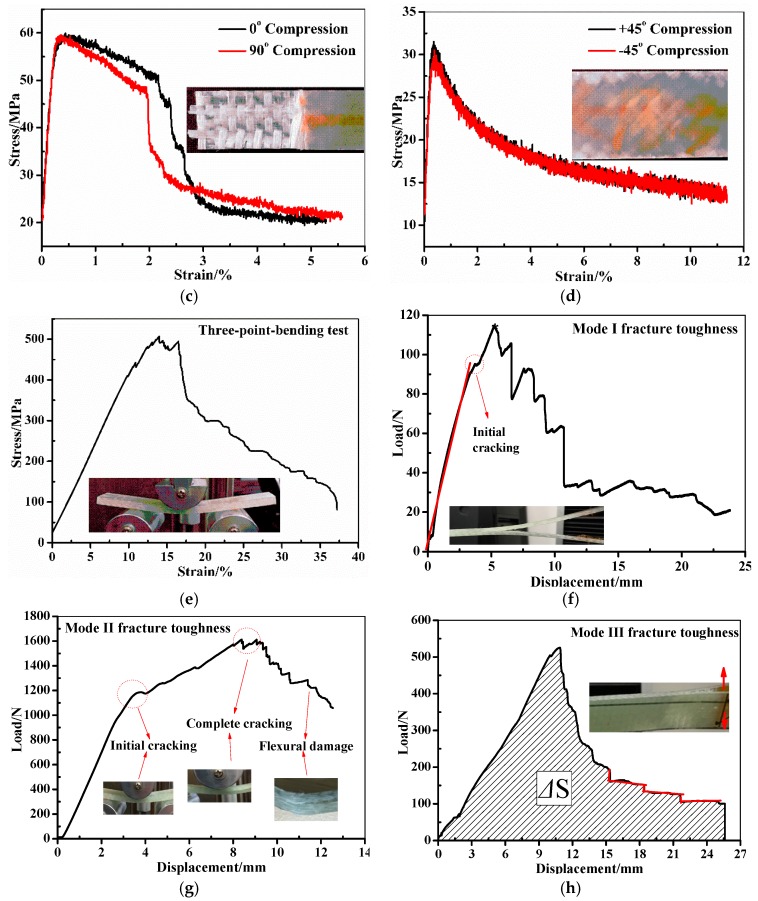
Mechanical performance of woven glass (WG)/epoxy composite laminates obtained in different experimental tests. (**a**): 90°/0° tensile; (**b**): 45° tensile; (**c**): 90°/0° compression; (**d**): 45° compression; (**e**): Three-point-bending test; (**f**): Mode-I fracture toughness test; (**g**): Mode-II fracture toughness test; (**h**): Mode-III fracture toughness test.

Figure 2 demonstrates the average stress-strain and load-displacement curves of the WGF/epoxy composite laminates in different mechanical tests. In detail, Figure 2a–d are the tensile and compression curves along 90°, 0°, and ±45° of the fibers, respectively. Figure 2e is the three-point bending test curve, while Figure 2f–h are the load-displacement curves obtained in DCB, 3ENF and the Mode III test, respectively. The elasticity, strength, and fracture toughness parameters together with their standard deviations of the WGF/epoxy composite laminates determined from Figure 2 are listed in Table 3, Table 4 and Table 5. In these tables, the longitudinal and transverse elastic modulus *E*_11_/*E*_22_, and the shear modulus *G*_12_ were calculated using the initial tensile curve slope in Figure 2a–d. The longitudinal/transverse tensile strength (*X_t_*/*Y_t_*), compression strength (*X_c_*/*Y_c_*), and the shear strength *S* were the peak stresses in Figure 2a–d that were determined by the maximum load divided by the cross-section area of the longitudinal, transverse, and 45° tensile specimens, respectively. The elastic modulus *E*_11_, *E*_22_ and *G* can be calculated by the following equation:

(1) $$E=\frac{S^{3}m}{4bh^{3}}$$

where *E* is the modulus, and *S* is the support span. *m* is the slope of the load-displacement curve, while *b* and *h* are the width and thickness, respectively. The following three formulas were adopted to calculate the Mode I, Mode II, and Mode III interlaminar fracture toughness (ILFT) of the WGF/epoxy composite laminates according to Figure 2f–h:

(2) $$G_{Ⅰc}=\frac{3P_{c}\delta_{c}}{2ba}$$

(3) $$G_{Ⅱc}=\frac{9{a_{0}}^{2}P_{c}\delta_{c}}{2b(\frac{3}{8}l^{3}+3{a_{0}}^{3})}$$

(4) $$G_{Ⅲc}=\frac{\Delta S}{2b\Delta a}$$

where, $G_{Ⅰc}$, $G_{Ⅱc}$ and $G_{Ⅲc}$. are the interlaminar fracture toughness; *a*_0_ and *a* are the pre-crack length and crack propagating length, respectively. *P_c_* and *δ_c_* are the load and displacement when the crack propagates to a specific length Δa, while *b* and *l* are the width and length of the specimens, respectively. Δ*S* is the area covered by *P_c_* and *δ_c_*, as indicated in Figure 2h. The minor deviations of experimental results in Table 3, Table 4 and Table 5 may be caused by uncertainty factors in the manufacturing process of the anisotropic composite materials [28,29]. Specially, in Table 5, the maximum loads in the three tests are 112.5 N, 1628.4 N, and 512.3 N, with corresponding failure displacements of 5.45 mm, 8.78 mm, and 10.67 mm, respectively. The ILFT of WGF/epoxy specimens is 1.65, 2.71, and 1.22 kJ/m^2^, which can be used to evaluate the interface performance of WGF/epoxy composite laminates in the following work.

### 2.3. Lamb Wave Inspection System

**Figure 3:**
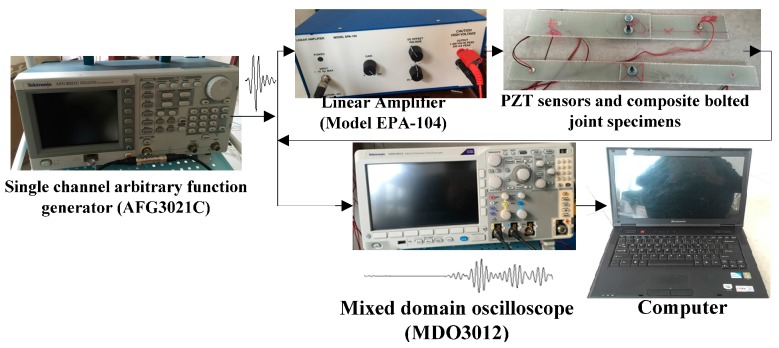
Experimental setup of the Lamb wave-based Structural health monitoring (SHM) system.

Figure 3 shows the experimental setup for Lamb wave propagation in the composite bolted joints in Case A and Case B. In the setup, a single channel arbitrary function generator (AFG3021C, Tektronix, INC., Beaverton, OR, USA) was adopted to excite the transmitting transducers. The actuating signal consisted of a 3.5-cycle sinusoidal tone burst. The signal had a frequency of 210 kHz and was modulated by a Hanning window. The actuating signal was then divided into two groups: one group was used as the input wave into the composite bolted joints after being amplified 20 times by a linear amplifier (Mode EPA-104, Piezo Systems, INC., Cambridge, MA, USA), while the other group was collected directly by the oscilloscope (MDO3021, Tektronix, INC., Beaverton, OR, USA). The signals were then transferred to a PC for data processing through a general purpose interface bus (GPIB) interface. It should be noted that the transmitting transducers adopted in the experiments were piezoelectric (PZT) wafers, and they had a circular shape with a diameter of 10 mm, and a thickness of 1 mm. All PZT transducers were bonded on the surface of the composite bolted joints using cyanoacrylate adhesive. Two PZT wafers were tied symmetrically on both ends of the joint specimen with an actuator-sensor distance of 215 mm, thus the signals had to travel through the joint region.

To investigate the pre-tightening force effect of the steel bolt on the wave propagation behavior in the composite joints, we applied the torque from 20 to 30 N·m on the joints using a digital display torque wrench. The wave signals in the two cases with different torques were collected by the system in Figure 3. The signals were recorded with a torque incremental interval of 1 N·m. All the obtained data were compared and analyzed in terms of wave amplitude. To evaluate the relationship between the waveform and the applied load on the composite bolted joints, we performed tensile tests on the joint specimens. During the tensile test, the load-displacement curves were collected, and the corresponding Lamb waves were captured. The load speed was also set as 2 mm/min at room temperature.

## 3. Fundamental Theory and Dispersion Feature of Lamb Wave in Composite Laminates

Lamb wave has a nature of dispersion and a multi-mode phenomenon, and it generally has symmetric (*S*) and antisymmetric (*A*) modes during propagation [8]. In an isotropic medium, the constituent properties, geometry, wave direction, and frequency could affect the Lamb wave modes [11]. Meanwhile, the wave modes may change when they meet a structural boundary and/or defects. However, due to anisotropic properties, wave propagation behavior in multi-layered composite laminates is very complex. The wave interactions depend upon not only the individual constituents but also their interface properties. In an *N*-layered composite laminate, there are six wave modes in total that could define a single guided wave mode in laminated composite plates [12]: two quasi-longitudinal (*L*), two quasi-shear vertical waves (*SV*), and two quasi-shear horizontal waves (*SH*). The Lamb wave can be generally described using its displacement field by satisfying Navier’s displacement equations within each layer [30], as following:

(5) $$\mu^{n}\nabla^{2}\mu^{n}+(\lambda^{n}+\mu^{n})\nabla (\nabla \cdot \mu^{n})=\rho^{n}\frac{\partial^{2}\mu^{n}}{\partial t^{2}}$$

where, $\rho^{i}$ and $\lambda^{i}$, $\mu^{i}$ are the density, Lame constant and displacement for the *i*th layer, respectively.

**Figure 4:**
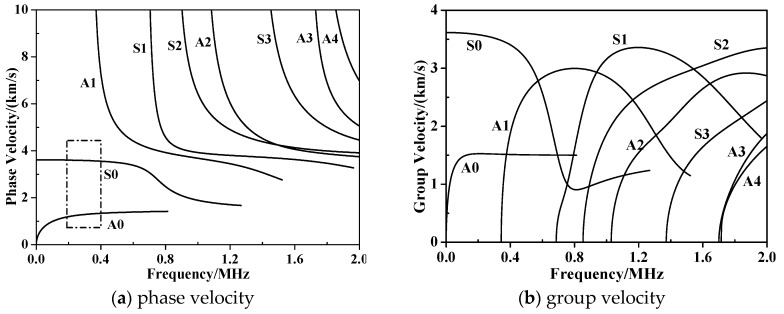
Dispersion property of WGF/epoxy composite laminates with a stacking sequence of [90°/0°]_4_.

The selection of the appropriate Lamb wave mode and frequency could deeply affect the wave structure analysis processing due to the dispersive nature. This is further complicated during the propagation into anisotropic material due to the complex structural features. To find out the suitable frequency for effectively monitoring the composite bolted joint, we calculated the dispersion characteristics by solving Equation (5) in Disperse software [31]. Figure 4 depicts the phase/group velocity-frequency curves of a boundary-free WGF/epoxy composite laminated with a stacking sequence of [90°/0°]_4_. The dispersion curves in Figure 4 clearly displayed the symmetric (*S*) and antisymmetric (*A*) Lamb wave modes. As highlighted with a dotted rectangle in Figure 4, a less dispersive region existed in the low frequency range where the *S0* and *A0* modes travelled at almost constant velocities. This frequency is referred to as the non-dispersion region. In detail, there were only *A0* and *S0* wave modes when the excited frequency was less than 400 kHz. In the following work, we selected the incident wave with a frequency of 210 kHz to monitor the health status of the composite bolted joints. This selected frequency could simplify the waveform analysis in post-processing since it could minimize the amplitude of unwanted mode signals.

## 4. Experimental Works on SHM of the Composite Bolted Joints Using Lamb Wave

### 4.1. Lamb Wave in Composite Bolted Joints Before Tensile Test

**Figure 5:**
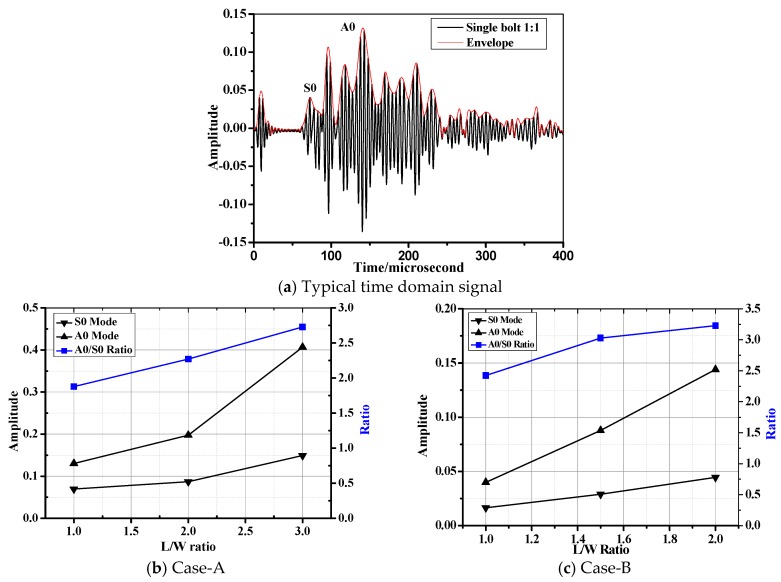
Waveform characteristics and the amplitude tendency in different composite bolted joints without tensile load (the applied torque on the bolts in the joints is 20 N·m).

Figure 5a–c depict the typical Lamb wave, *A0*/*S0* amplitude and their ratio tendency in the composite bolted joints, respectively. It should be noted that the tested specimens in Figure 5a are in the free boundary condition without tensile load. For easy observation, we gave the incident Lamb wave and the reception signals by the actuator and sensor pairs, simultaneously. The received waves were formed by different sources such as direct wave, the inner scatter, and boundary reflection waves. In Figure 5a, the propagation duration of the direct Lamb wave in different cases matched the *S0* mode in the dispersion curves in Figure 4. The predicted phase/group velocity was calculated by dividing the actuator-sensor distance (215 mm) by the arriving time; this was 3700 m/s. The first wave package was the *S0* mode Lamb wave that propagated directly from the actuator to the sensor, and its group/phase velocity was the largest. According to the dispersion curve, the phase/group velocity of the *A0* Lamb wave was 1500 m/s, thus the wave package with arriving times approximately of 140 µs was the *A0* mode Lamb wave package, as marked in Figure 5a. There are normally two wave packages between *A0* and *S0* wave modes, which are formed by Lamb wave scattering effect such as reflection, transmission and mode conversion when they meet the discontinuity in the overlapped joint region. As shown in Figure 5b,c, the amplitude of the *S0* mode was smaller than that of *A0* mode in both cases. This was mainly the case because that *S0* and *A0* mode are excited differently, depending on the tone burst excitation frequency [32,33,34]. Furthermore, since it is difficult for in-plane particle motion to cross the interface, *S0* mode is mostly retained in the plate, while partial energy of the out-of-plane *A0* modes will leak into the interface. Another aspect, we found that the Lamb wave amplitude increased with increasing of *L*/*W* ratio. This phenomenon can be explained as following: In the joints, the discontinuity occurs at the ending edge and the bolt region. This leads to a result where the incident Lamb wave will be partially received by the sensor, and partially reflected. Since the overlap area increased, the Lamb wave energy during its propagation in the joints with higher *L*/*W* ratio also increased. This mechanism further leads to the larger wave amplitude. Moreover, the amplitude in Case A (Figure 5b) was relatively larger than that of Case B (Figure 5c), which was mainly due to the increased number of fastener holes that could dissipate the wave energy by reflection.

**Figure 6:**
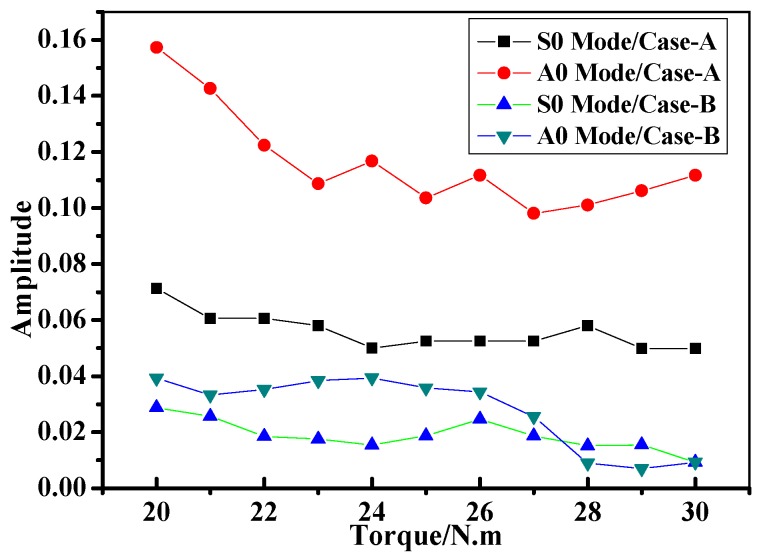
Amplitude of *S0* and *A0* mode as the function of the applied torque on the bolt.

Also, the interface between two jointed composite plates could affect the wave energy and cause the wave amplitude decline by reflecting and scattering during its propagation. As known, in the composite bolted joints, the interface state between the two jointed specimens can be evaluated by the torque on the bolts during the assembling process. In order to evaluate the interface bonding effect on Lamb wave behavior in composite bolted joints, Figure 6 describes the relationship between the amplitude of *S0* and *A0* mode waves and the torque on the bolts in different composite joints cases. As shown in the figure, the amplitude of both the *S0* and *A0* mode waves showed an approximately decreasing tendency with an increase of torque. The wave amplitude in Case A was generally larger than that of Case B. As discussed, this was attributed to the wave reflection phenomenon in Case B being more severe than that of Case A. To make signal analysis processing easier, we applied a torque of 20 N·m on all the bolts, since their *S0* and *A0* wave amplitudes were easier to distinguish among all the complex wave packages in the received signals.

### 4.2. Lamb Waves Inspection of the Composite Bolted Joints after Tensile Test

**Figure 7:**
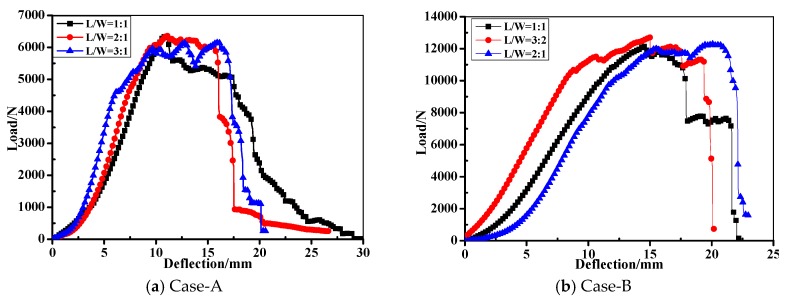
Typical load-deflection curves of the composite bolted joints in tensile test.

**Figure 8:**
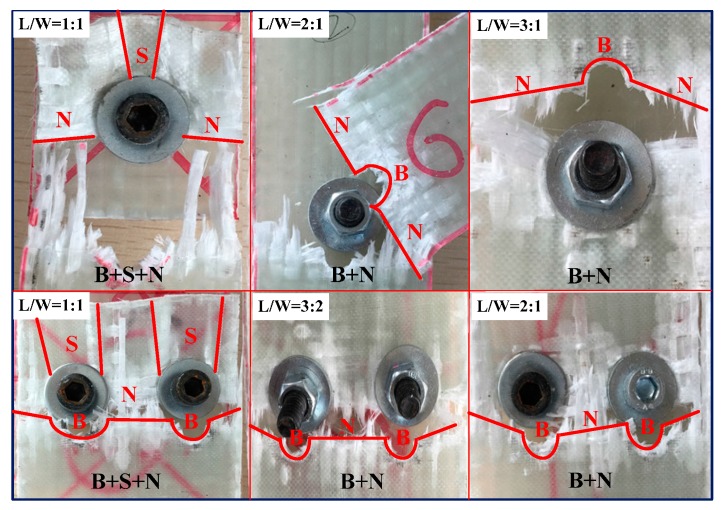
The final damage modes of the composite bolted joints after the tensile tests (In this figure, *N* indicates net-tension, *S* indicates shear out, and *B* means bearing failure mode).

Figure 7 illustrates the representative curves of macroscopic load-deflection (*L*-*D*) of the composite bolted joints in tensile test. It can be seen from the experimental results that the tensile load appeared initially to be an approximately linear relationship, and with plastic yielding with increased displacement. The failure loads were approximately 6300 N and 12,000 N for Case A and Case B, respectively. In terms of joint configuration, the potential failure modes in bolted composite joints are comprised of cleavage (*C*), net-tension (*N*), shear out (*S*), and bearing (*B*) [16,18]. Figure 8 shows the final damage pattern after the experiments. Since the strength of the steel bolt is much higher than that of the WGF/epoxy composites, composites damages were the main failure modes. Damages occurred on L/W=1:1 bolted joints are the comprehensive failure modes that comprised of *B* + *S* + *N*, while the other specimens appeared for the failure mode as *B* + *N*. It is worth mentioning that during the test, the occurring sequence of failure mode in the composite plate was *B* > *S* > *N*. In the tensile test, the tensile stress in the joints was mainly supported by the shear load from the used bolts, which further limited the tensile peak load in Figure 7. This stress firstly caused bearing failure (*B*) around the bolt hole, and then shear damage appeared. It should be noted that this process was accompanied by other failures such as delamination and fiber/matrix cracking. These failure modes in the composite panel further led to the final failure mode (*N*) on the specimens. Since fiber breaking in the composite specimens progressively occurred, it resulted in a ‘load plateau’ in the load-displacement response. Both the failure process and the final damage modes of the composite bolted joints deeply affected the captured Lamb wave feature in the Lamb wave inspection procedure, and discussions on this aspect will be carried out in the following work.

**Figure 9:**
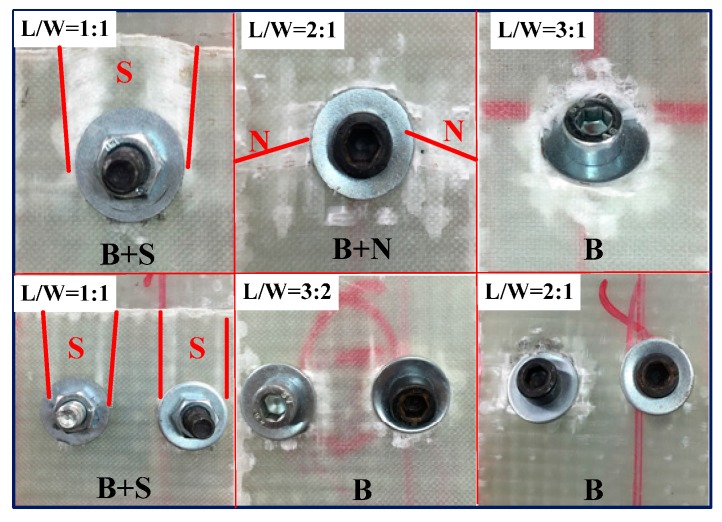
Damage modes of the composite bolted joints before completely failure after tensile test (In this figure, *N* indicates net-tension, *S* indicates shear out, and *B* means bearing failure mode).

**Figure 10:**
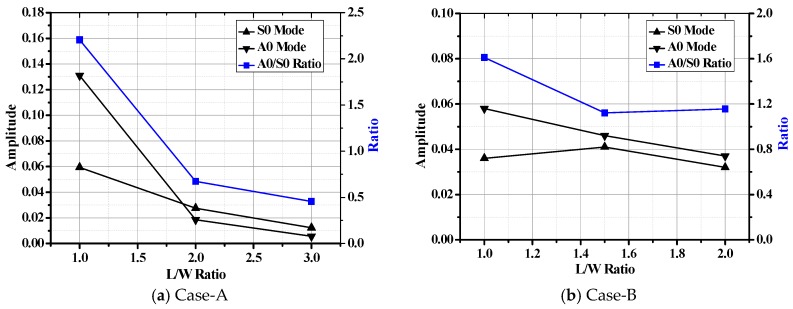
Waveform characteristics and the amplitude tendency in different composite bolted joints after tensile load.

Because the composite bolted joints after the tensile test were completely discontinuous and the Lamb wave could not be captured by PZT sensors the SHM processing, we selected specimens over 5 kN and 10 kN tensile loads as research objects to establish quantitative connections between the extracted signal characteristics and the damage parameters. As concluded from the *L*-*D* curve in Figure 7, these applied loads were very close to the failure tensile load of the joints. The corresponding damage modes of the specimens after these tensile loads are illustrated in Figure 9. Unlike the final damage modes in Figure 8, the damage modes are mainly comprised of bearing failure mode accompanied with some shear out, and net-tension failure modes. The *A0/S0* wave amplitude and their ratio tendency after the tensile load are presented in Figure 10. It should be noted that the wave was excited and received by the PZT actuator-sensor pairs whose locations were the same as the specimens in Figure 5. In contrast to Figure 5, the amplitude in Figure 10 showed a decline in tendency with an increase in *L/W* ratio.

### 4.3. Lamb Wave Inspection of the Composite Bolted Joints during Tensile Test

**Figure 11:**
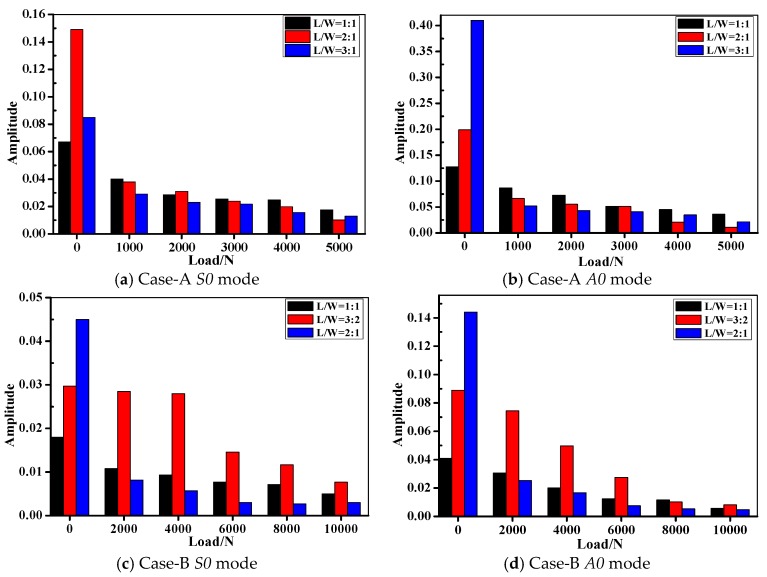
Comparison between amplitude of composite bolted joints during the tensile tests.

**Figure 12:**
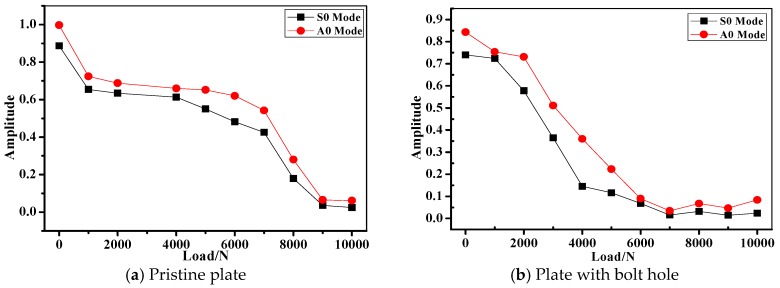
Amplitude of *S0* and *A0* mode of a pristine and drilled composite plate during the axial tension test.

Figure 11 shows the amplitude of *S0* and *A0* mode as the function of applied load in different joints during the tensile test. Clearly in the figure, the amplitude showed a declining tendency with the increase of the applied tensile load. Taking the Lamb wave captured in Case A as the example, the amplitude of *A0* mode was larger than that of *S0* mode when the tensile load was applied at the same level. As discussed above, this phenomenon is associated with the unique wave propagation characteristics of *A0* and *S0* mode in the composite plates. Differences between Lamb wave amplitudes of unloaded joints are very easy to distinguish between different *L*/*W* specimens, while the *S0*/*A0* amplitude was nearly the same when the joints were under tensile load. In terms of the joints in Case B, it seemed that the wave amplitude in *L*/*W* = 3:2 specimens was always larger than the other cases under tensile load. The amplitude decreasing phenomenon could be interpreted as the following: As it is known, the Lamb wave propagation characteristics vary with entry angle, frequency and structural geometry [35,36]. Compared the *L*-*D* curve in Figure 7 and the damage modes in Figure 9, the damage modes under consideration were mainly comprised of bearing failure, whose severity is proportional to the applied tensile load. With the increasing of tensile load, the waveforms changed with the varying geometries. Moreover, since tensile strength on the composite bolted joints is offset axial tension, the contact between the two plates in the joint region began to weaken. As a result, much less kinetic energy of from the wave particles could go through the joints region and received by the PZT sensor. In order to verify this assumption, we performed the axial tensile experiment on a pristine composite plate and a composite plate with a bolt hole (drilled plate). Figure 12 shows the amplitude tendency of the two specimens as the function of the applied load. With the tensile load increasing from 0 to 10,000 N, the *S0*/*A0* mode amplitudes declined from 0.887/0.997 to 0.0243/0.0611 for the pristine plate in Figure 12a, while the *S0*/*A0* mode amplitudes declined from 0.74/0.843 to 0.024/0.0838 for the drilled plate in Figure 12b. The wave amplitudes show a decreased tendency with the increasing of tensile load, which was due to the increased geometry length of the specimens. Another aspect, the amplitude attenuation level in Figure 11 was sharper than that of Figure 12. By comparison, this was mainly attributed to the weakened contact property of the composite plate in the joint region.

## 5. Simulation Works on Lamb Waves in Composite Bolted Joints

### 5.1. 3D FE Model Description

**Figure 13:**
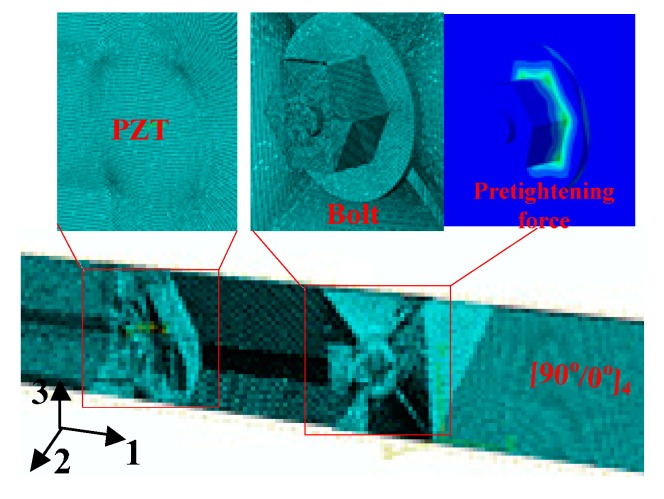
FE model in the Lamb wave inspection simulation with details (Coordinate system: 1: length, 2: thickness and 3: width direction).

As discussed in the experimental section, the Lamb wave propagation phenomenon in the joint region is very complex, mainly due to structural discontinuity. In order to further interrogate the joint influence on the Lamb wave propagation feature, FE simulations were conducted. For the sake of brevity, the main aim of the performed FE simulation was to study the Lamb wave propagating feature in *L*/*W* = 1:1 composite bolted joints in Case A. In [37], we carried out the simulation work on woven composite laminates under low-velocity impact load. Also based on the developed three-dimensional FE method in previous work [38], we adopted 3D Hashin criteria and a user material subroutine to perform the Lamb wave inspection on the composite bolted joints. The simulation was carried out on the Abaqus/Explicit (Version: Abaqus 6.12, Simulia, Providence, RI, USA) platform, and the FE model with details is shown in Figure 13. The bolted composite laminates had four layers, each layer having a thickness of 0.55 mm. The fiber stacking sequence was [90°/0°]_4_, and the final dimension, bolt location in the model was the same as the experiment in Figure 1 and Table 1. The laminates were assigned anisotropic properties according to the parameters listed in Table 3, Table 4 and Table 5. For the interface property in the overlapped jointed region, ‘hard contact’ was applied between the two parts. In terms of the interlaminar property between each composite layer, the surfaces were tied together in the interaction module in Abaqus. The element type was an eight-node C3D8R with a spatial resolution of 0.1 mm. This is more than ten times smaller than the smallest wave length of the Lamb waves in the considered frequency range [35,39]. For the excitation signal, a 3.5 cycle Hanning-windowed sinusoidal tone burst was excited by a circular fine mesh region with diameter of 10 mm. In order to keep pace with the experiment, two steps were performed in the simulation: a pre-tightening force on the bolt was applied in the first step, and then the Lamb wave propagating behavior in the bolted joints was carried out in the second step.

### 5.2. Simulation Results

**Figure 14:**
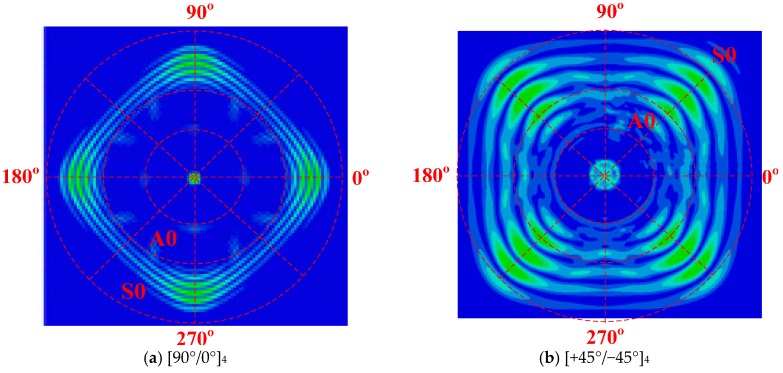
Slowness profiles of *S0* and *A0* modes in WGF/epoxy composite laminates with different ply modes.

Unlike Lamb waves in isotropic plates, the wave velocity was subject to the propagation direction in non-isotropic materials [40,41]. Therefore, before FE simulations on composite bolted joints were carried out, we firstly investigated the slowness profiles of [90°/0°]_4_ and [+45°/−45°]_4_. Figure 14 described the measured velocities of the *S0* and *A0* modes in the composite laminates (dimension: 800 × 800 × 2 mm^3^). We can found that the two Lamb wave modes travelled at distinct velocities in different directions. Taking [90°/0°]_4_ composite plate as the example, the *S0* and *A0* wave velocities along 45° fiber direction were the lowest among all the directions. This phenomenon matched the wave propagating theory in laminated composite panel in Equation (5), and can be explained as following: The woven fabrics in the WGF/epoxy composite laminates are along 90°/0°, and the strength and modulus along the fiber direction was much higher in the composites. The particle elastic motions of Lamb wave in the fiber direction were stronger and this led to a high wave amplitude and velocity in the slowness profiles. It should be noted that the Lamb waves in the joints followed the same slowness profiles in Figure 14a. Since the sensor-actuator path was along the fiber direction (0° in slowness profile), the captured Lamb wave signal had a higher amplitude and velocity in the [90°/0°]_4_ composite bolted joints.

**Figure 15:**
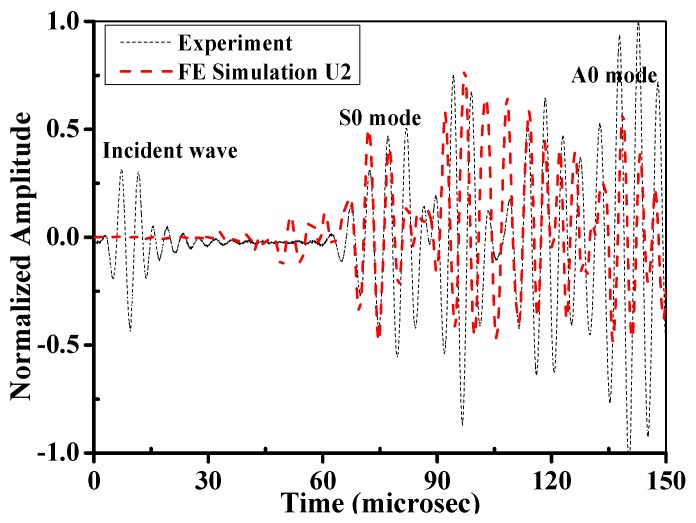
Comparison of the normalized amplitude between FE simulation and the experiments.

**Figure 16:**
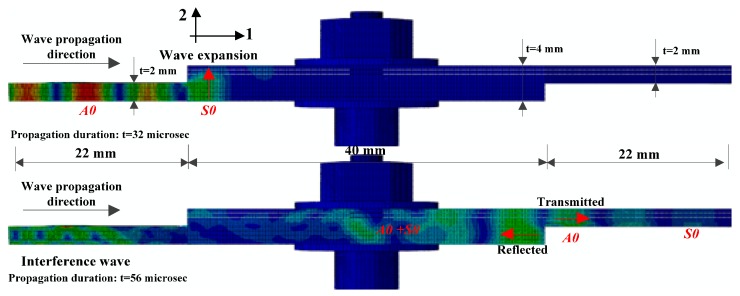
Displacement fields (U2) of Lamb waves in the composites bolted joints for an excitation frequency of 210 kHz obtained in the FE simulations.

The wave structures of the composite bolted joints in the simulation and experiment are compared in Figure 15. Because the wave is mainly propagated by particle motions along the thickness direction, we used the U2 displacement fields to represent the Lamb wave in the simulation. It was obvious that the *S0* mode was predicted successfully, and the simulation and experimental results matched well. Like the received signal in experiments, two wave packages were found between *S0* and *A0* in the simulation. Minor differences between the simulation and the experimental results of the *A0* amplitude were attributed to the fact that the elastic vibration of *A0* wave at the composite plate edges in the experiment is difficult. Meanwhile, the distortion of the *A0* wave packets due to dispersion could also have led to the differences observed. As calculated by the relationship between propagation duration and the sensor-actuator distance in the FE, the predicted *S0*/*A0* wave velocity followed the dispersion curves in Figure 4. Figure 16 shows the Lamb wave propagation feature when it propagates through the joint region. As can be seen, the fundamental *S0* and *A0* modes were captured well, and their propagation duration was consistent with the signal curve in Figure 15. It is worth mentioning that two significant phenomena were found in Figure 16: Expansion of the excited Lamb waves occurred at the incident edge of the jointed plate, and when it was propagating from a large cross-section to a small area at the second edge, reflection and transmission occurred. This phenomenon implies that the wave motion in the composite bolted region was constructed during the propagation.

## 6. Conclusions

This paper investigated Lamb wave-based structural health monitoring technology on two types of composite bolted joints with different *L*/*W* ratios. As a preliminary study, the following conclusions can be summarized:

- Relationships between *S0*/*A0* wave and the failure mode of the composite bolted joints were found. Damages in the joints mainly affected the amplitude of the Lamb wave, and the difference in *S0/A0* amplitude could be adopted to identify the failure mode of the joints after tensile load.
- The amplitude of the Lamb wave varied with the applied torque on the joints. The *S0*/*A0* wave amplitudes showed a decrease in tendency with the increasing of tensile load, and this was mainly caused by the increased specimen length and the weakened contact property in the joint region during the tensile test.
- The built 3D FE model could capture the slowness profiles of Lamb waves well when they were propagating in multi-layered composite laminates. The captured wave in the simulation matched the experiment results, and wave expansion and reflection phenomenon were observed in the overlapped joint region.

## Figures and Tables

**Table 1:** Structural style and geometric dimension of the two studied composite bolted joints in the experiment.

Parameter	Case A			Case B		
W	40	40	40	60	60	60
L	40	80	120	60	90	120
L/W	1:1	2:1	3:1	1:1	3:2	2:1
L_total_	450	410	380	450	410	380
Bolt location	(L/2, W/2)	(L/2, W/2)	(L/2, W/2)	(L/2, W/4)	(L/2, W/4)	(L/2, W/4)

**Table 2:** Test standard and dimension of woven glass fabric (WGF)/epoxy in different experiments.

Test Method	ASTM Standard	Dimension (*L* × *W* × *T*, mm^3^)	Calibrating Length (mm)
90°/0° tensile	D3039/D3039M-08	220 × 20 × 2.5	140
±45° tensile	D3518/D3518M-13	180 × 20 × 2.5	100
±45°/90°/0° compression	D3410/D3410M-95	160 × 20 × 2.5	80
DCB	D5528-13	220 × 25 × 2.5	25 (precrack)
3ENF	D7905/D7905M-14	100 × 25 × 2.5	80
Mode III	E1922-04	100 × 25 × 2.5	25 (precrack)

ASTM: American Society for Testing Materials; DCB: double cantilever beam; 3ENF: three-point end notched flexural.

**Table 3:** Elastic parameters obtained in the mechanical test.

*E*_11_/GPa	*E*_22_/GPa	*E*_33_/GPa	*G*_12_/GPa	*G*_13_/GPa	*G*_23_/GPa	*µ*_12_	*µ*_13_	*µ*_23_	*ρ*/(g/cm^3^)
11.8 ± 0.23	11.8 ± 0.25	0.58 ± 0.02	4.82 ± 0.08	4.82 ± 0.08	4.82 ± 0.08	0.05 ± 0.01	0.24 ± 0.01	0.23 ± 0.01	1.65

**Table 4:** Strength parameters obtained in the mechanical test (MPa).

*X*_T_	*X*_C_	*Y*_T_	*Y*_C_	*Z*_T_	*Z*_C_	*S*_12_	*S*_13_	*S*_23_
485.05 ± 12.54	59.94 ± 2.37	488.91 ± 15.29	59.58 ± 1.27	52.14 ± 0.98	180.24 ± 1.89	108.4 ± 5.34	108.4 ± 5.34	108.4 ± 5.34

**Table 5:** Interlaminate parameters of WGF/epoxy composites calculated from fracture toughness tests.

Test Method	Maximum Load/N	Failure Displacement/mm	Strain Energy Release Rate/(kJ/m^2^)
Mode-I	112.5 ± 3.24	5.45 ± 0.05	1.65 ± 0.05
Mode-II	1628.4 ± 20.05	8.78 ± 0.32	2.71 ± 0.03
Mode-III	512.3 ± 4.34	10.67 ± 1.12	1.22 ± 0.01
